# The prognostic and clinicopathological significance of PD-L1 expression in patients with diffuse large B-cell lymphoma: a meta-analysis

**DOI:** 10.1186/s12885-019-5466-y

**Published:** 2019-03-27

**Authors:** Liping Qiu, Hanlu Zheng, Xiaoying Zhao

**Affiliations:** Department of Hematology, The Second Affiliated hospital of Zhejiang University School of Medicine, Hangzhou, 310009 China

**Keywords:** Diffuse large B-cell lymphoma, Programmed cell death receptor 1 ligand 1 (PD-L1), Prognosis, Meta-analysis

## Abstract

**Background:**

Programmed cell death receptor 1 ligand 1 (PD-L1) expression in various tumors, including hematologic malignancies, has recently become a research topic of great interest. We performed a meta-analysis to evaluate the prognostic and clinicopathological value of PD-L1 expressed in tumor cells of patients with diffuse large B-cell lymphoma (DLBCL).

**Methods:**

Relevant studies were identified from PubMed, EMBASE, Web of Science and the Cochrane Library. The hazard ratio (HR) and 95% confidence interval (95% CI) were used for analyzing survival outcomes, and the odds ratio (OR) was used for analyzing clinicopathological parameters.

**Results:**

Pooled results showed that tumor cell PD-L1 expression is associated with poor overall survival (OS) (HR = 2.128, 95% CI: 1.341–3.378, *P* = 0.001), the non-germinal center B-cell-like subtype (OR = 2.891, 95% CI: 2.087–4.003, *P* < 0.000), high international prognostic index score (3–5) (OR = 1.552, 95% CI: 1.111–2.169, *P* = 0.010), B symptoms (OR = 1.495, 95% Cl: 1.109–2.015, *P* = 0.008), positive MUM1 expression (OR = 3.365, 95% Cl: 1.578–7.175, *P* = 0.002) and negative BCL6 expression (OR = 0.414, 95% Cl: 0.217–0.792, *P* = 0.008). Sensitivity analysis showed that there was no publication bias among these studies.

**Conclusions:**

Our meta-analysis supported the idea that tumor cell PD-L1 expression may represent a promising biomarker for predicting poor prognosis and is associated with adverse clinicopathologic features in DLBCL patients.

## Background

Diffuse large B-cell lymphoma (DLBCL) is the most common subtype of non-Hodgkin lymphoma (NHL), accounting for approximately 30–40% of newly diagnosed NHL cases [1]. Due to its highly heterogeneous features, approximately 35% of DLBCL cases cannot benefit from rituximab (R) combined with anthracycline-based chemotherapies and eventually experience relapsed/refractory disease [2]. Therefore, it is critical to identify additional biomarkers and new therapeutic targets for DLBCL.

The programmed cell death receptor 1/programmed cell death receptor 1 ligand 1 (PD-1/PD-L1) pathway regulates effector T-cell responses and protects tissues from immune-mediated damage [3]. However, mounting evidence shows that activation of the PD-1/PD-L1 pathway allows tumors to elude host immune surveillance [2, 4]. PD-L1 is a member of the B7 family, which is expressed on the surface of antigen-presenting cells and tumor cells [3]. Evidence has suggested that aberrant PD-L1 expression is associated with poor prognosis. Monoclonal antibody blockade of the PD-1/PD-L1 pathway demonstrated clinical activity in several types of cancers, such as non-small cell lung cancer [5, 6], melanoma [7], renal cell carcinoma [8, 9], and hematological malignancies including relapsed/refractory classic Hodgkin lymphoma (HL) [10]. However, the prognostic role of PD-L1 expression in DLBCL remains elusive. Different research targets such as tumor cells, tumor-infiltrating nonmalignant cells [11] or soluble PD-L1 [12] produce different results. Accordingly, we conducted a meta-analysis to explore whether the tumor cell expression of PD-L1 correlates with the clinicopathological features and prognosis of patients with DLBCL.

## Methods

### Search strategy

This meta-analysis was reported according to the Preferred Reporting Items for Systematic Reviews and Meta-Analyses statement [13]. We searched PubMed, EMBASE, Web of Science and the Cochrane Library databases for articles published before December 31, 2018. The terms employed for our search included “PD-L1”, “PDL1”, “B7-H1”, “CD274”, “programmed death ligand 1”, “diffuse large B-cell lymphoma” and “DLBCL”. The language of publications was restricted to English. To identify additional studies, review of the reference lists of relevant articles was also performed.

### Selection criteria

Studies were selected if they met the following requirements: (1) Patients were diagnosed with histologically confirmed DLBCL; (2) Studies used immunohistochemistry (IHC) as a measurement technique and had a definite cutoff for determination of PD-L1 positivity; (3) Studies provided the associations of PD-L1 expression with overall survival (OS)/ progression-free survival (PFS) and clinicopathological features of DLBCL; and (4) The hazard ratio (HR) and its 95% confidence interval (CI) were provided in terms of direct extraction, the Kaplan-Meier Curve from which the HR was calculated or the *P* values and the numbers of the outcomes using Parmar’s method [14]. The exclusion criteria included the following: (1) The study was an animal study or cell line study, or the publication was a letter, conference abstract, expert opinion, review or included results with unavailable full text; (2) Studies did not evaluate PD-L1 expression in DLBCL patients; (3) Targets were restricted to tumor-infiltrating nonmalignant cells or soluble PD-L1 expression in DLBCL patients; (4) The data for estimating the HR and 95% CI were insufficient; (5) Patients had received anti-PD-1/PD-L1 therapy; (6) The cutoff value of positive PD-L1 expression was not reported or reported only as inaccurate data; or (7) The Newcastle–Ottawa quality assessment scale was less than 6 [15].

### Data extraction and statistics

Two reviewers independently examined the included studies and extracted the relevant data. The following data were extracted: the name of the first author, the year of publication, country of origin, cut-off value used, antibodies used, the number of positive or negative cases, follow-up time, HRs and their 95% CIs of OS or PFS and clinicopathological parameters.

We pooled HRs and their 95% CIs to analyze the prognostic significance of PD-L1 expression on the survival outcome of DLBCL. If the HRs and their 95% CIs were not reported directly, data were extracted from the Kaplan-Meier survival curves published in the article and estimated using Engauge Digitizer version 4.1(http://markummitchell.github.io/engauge-digitizer/) or the *P* values and the numbers from the outcomes. In addition, we pooled odds ratios (ORs) and their 95% CIs to analyze the association between PD-L1 expression and clinicopathological parameters. Statistical heterogeneity was evaluated by a chi-squared test and *I*^*2*^. Significant heterogeneity was defined as *I*^*2*^ > 50% or *P* value< 0.1. If heterogeneity was observed, we used a random-effects model to calculate the combined effect sizes. If not, a fixed-effects model was employed. Sensitivity analyses were performed to estimate whether any individual study influenced the pooled HRs or ORs. Publication bias was assessed by Egger’s and Begg’s tests. All statistical analyses were performed using STATA version 12.0 (Stata Corporation, College Station, Texas, USA). All statistical tests were two-sided, and *P* < 0.05 was considered indicative of statistical significance.

## Results

### Search results

**Fig. 1:**
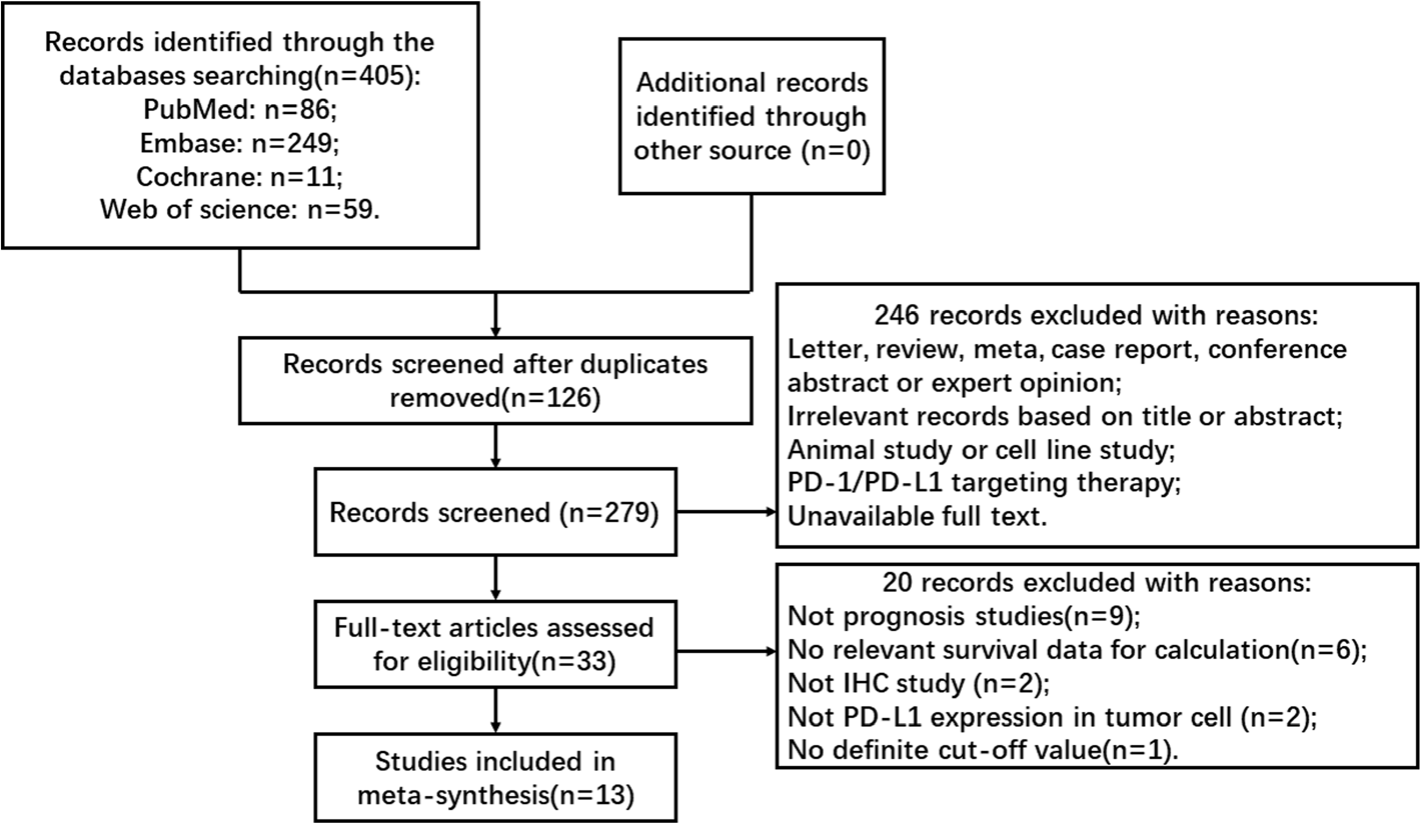
Flow chart of the selection procedure for relevant studies

After a search with the abovementioned retrieval strategy and removal of duplicates, a total of 286 potentially relevant articles were initially identified. After reviewing the titles and/or abstracts, 33 articles remained. According to the inclusion and exclusion criteria, 13 studies with 1613 cases of DLBCL were ultimately included in our meta-analysis [11, 16–27] (Fig. 1). Among these 13 articles, 12 studies provided HRs and 95% CIs of overall survival, while 6 studies provided HRs and 95% CIs of PFS. All studies were retrospective study designs and used immunohistochemistry to detect PD-L1 expression in tumor cells. The specific characteristics of the included studies are shown in Table 1. Cutoff was defined as the percent PD-L1 expression in tumor cells among the total cells from the pathological tissue. Five studies [16, 18, 24, 25, 27] evaluated PD-L1 positive expression as the proportion of tumor cells showing only membranous staining or only cytoplasmic staining, while others focused on membranous and/or cytoplasmic staining. Five studies [16, 20, 21, 26, 27] provided the association between PD-L1 and pathologic features. Xing et al. [16] and Shi et al. [27] adopted cutoff values for the high expression of Ki-67, MYC, BCL2 and BCL6 of 80, 40, 30 and 30%, respectively. However, Hu et al. [21] chose 90% as the cutoff value for increased levels of Ki-67 expression. Bledsoe et al. [20] and Kwon et al. [26] did not provide the definite cutoff values.

**Table 1:** Characteristics of enrolled studies in diffuse large B-cell lymphoma

author	country	year	cut-off	cases	antibody			Follow-up (month)	therapeutic regimen	HR (95%CI) of OS	HR (95%CI) of PFS
				PD-L1+/−	clone	dilution	source				
Kiyasu J	Japan	2015	0.3	34/239	EPR1161	1:200	Dako	60	80%RCHOP/CHOP-like	1.809 (1.051–3.112)	NA
Xing W	America	2016	0.3	14/72	E1L3N	NA	Cell Signaling	21 (0.067–175)	RCHOP	2.418 (1.031–5.670)	2.130 (1.050–4.330)
Kwon D	Korea	2016	0.1	77/49	E1L3N	NA	Cell Signaling	52 (16–165)	RCHOP	0.430 (0.152–1.217)	NA
Dong L	China	2016	0.05	54/46	ab153991	1:200	NA	52.4 (1.5–89.1)	CHOP/CHOP-like+ 39%R	4.740 (1.097–20.477)	NA
Four M	France	2016	0.05	12/20	SP142	NA	Ventana	17 (1–78)	chemotherapy and/or radiotherapy	7.7 (1.6–37.2)	1.7 (0.67–4.28)
Bledsoe JR	America	2016	0.25	20/26	E1L3N	1:200	Cell Signaling	78	RCHOP	0.29 (0.06–1.25)	0.19 (0.04–0.83)
Hu LY	China	2017	0.05	100/104	NA	1:50	Cell Signaling	52 (1–114)	RCHOP+ 26.7%RT	4.055 (1.610–10.230)	1.584 (1.000–2.510)
Fang X	China	2017	0.1	20/54	SP142	ready to use	ZSGB-BIO	2.4–86.4	CHOP/CHOP-like+ 55.2%R	2.547 (0.964–6.730)	NA
Liu Y	China	2018	0.3	11/81	SP142	NA	NA	58 (1–78)	RCHOP	4.63 (1.53–13.99)	NA
Ishikawa E	Japan	2018	0.05	3/48	SP142	NA	NA	42 (3.5–150)	86.4%RCHOP	5.72 (1.50–21.8)	NA
Sun C	China	2018	0.5	34/253	22C3	NA	Dako	76	CHOP/CHOP-like+ 53.7%R	1.494 (0.868–2.571)	NA
Kwon HJ	Korea	2018	0.3	23/84	E1L3N	1:100	Cell Signaling	NA	86.9%RCHOP/CHOP-like	1.21 (0.15–9.98)	2.21 (0.59–8.27)
Shi YF	China	2018	0.3	21/104	SP142	1:100	Ventana	25.7 (0.8–131.1)	58.1%RCHOP/CHOP-like;29.7%surgery	NA	0.379 (0.149–0.962)

*NA* not available; *RCHOP* rituximab, cyclophosphamide, doxorubicin, vincristine and prednisone; *RT* radiotherapy;*HR* hazard ratio;*CI* confidence interval;*OS* overall

survival; *PFS* progression-free survival

### Association between PD-L1 expression and OS

**Fig. 2:**
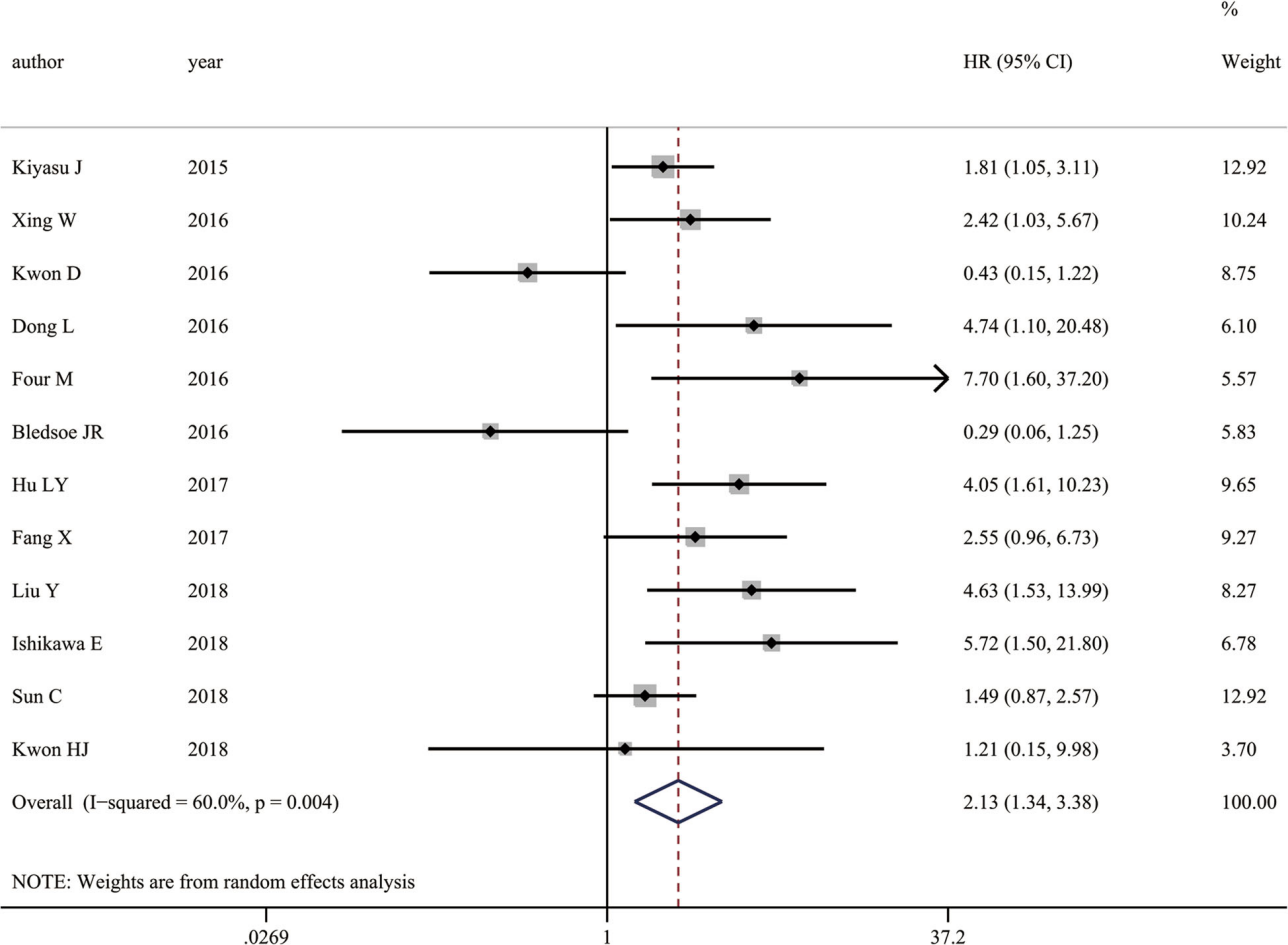
Forest plot describing the association between PD-L1 expression and OS with DLBCL

**Fig. 3:**
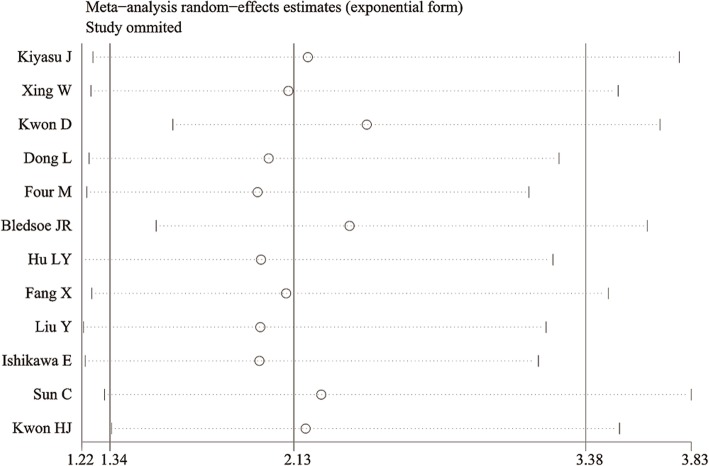
Sensitivity analysis of the relationship between PD-L1 expression and OS

We enrolled 12 studies to investigate the association between PD-L1 expression and OS in 1478 patients with DLBCL [11, 16–26]. Strong heterogeneity was observed (*I*^*2*^ = 60.0%, *P* = 0.004). Therefore, we used a random effects model and discovered that positive PD-L1 expression was associated with shorter OS compared with that of negative expression (HR = 2.128, 95% CI: 1.341–3.378, *P* = 0.001) (Fig. 2). Sensitivity analysis showed that the pooled HRs were not significantly affected by any single study (Fig. 3). For further evaluation, we performed a subgroup analysis of the cutoff value and region. The pooled subgroup results showed that studies using ≥30% (HR = 2.128, 95% CI: 1.341–3.378, *P<*0.000; I^2^ = 0, *P* = 0.44) as the cutoff value for predicting prognosis showed increased significance compares with those using<30% (HR =2.195, 95% CI: 0.884–5.446, *P* = 0.090; *I*^*2*^ = 74.5%, *P* = 0.001) (Table 2). Moreover, the predictive effects of PD-L1 became stronger when the studies were limited to Asian populations (HR =2.195, 95% CI: 1.352–9.980, *P* = 0.001; *I*^*2*^ = 56.2%, *P* = 0.019) or to Chinses populations (HR =2.729, 95% CI: 1.618–4.605, P<0.000; I2 = 37.1%, *P* = 0.174) (Table 2).

**Table 2:** Subgroup analysis of pooled hazard ratios for DLBCL with positive PD-L1 expression

Stratified analysis	No. of studies	No. of patients	HR	95%CI	*P*	Heterogeneity	
						*I* ^*2*^	*P*
cutoff							
<0.3	7	633	2.195	0.884–5.446	0.009	74.50%	0.001
≥ 0.3	5	845	2.128	1.341–3.378	0	0	0.440
region I							
Asian region	9	1314	2.195	1.352–9.980	0.001	56.20%	0.019
Non-Asian region	3	164	1.779	0.356–8.896	0.483	78.30%	0.010
region II							
China	7	757	2.729	1.618–4.605	0	37.10%	0.174
Others	5	721	1.620	0.755–3.479	0.216	69.10%	0.004

*HR* hazard ratio;*CI* confidence interval

### Association between PD-L1 expression and PFS

**Fig. 4:**
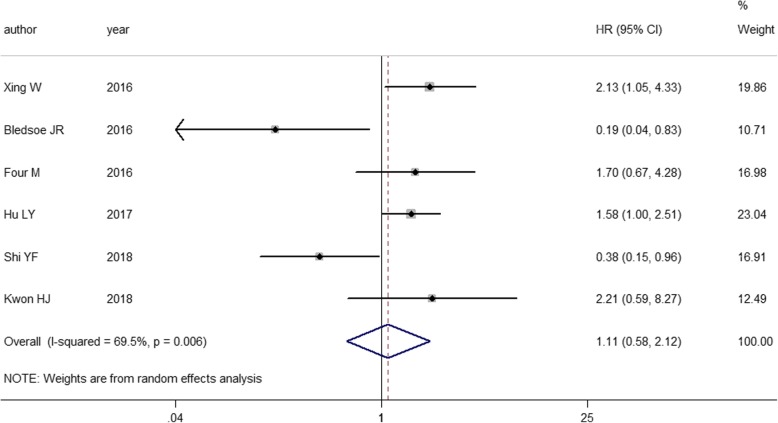
Forest plot describing the association between PD-L1 expression and PFS with DLBCL

Only 6 out of 13 studies including 600 patients with DLBCL provided information about PFS [16, 19–21, 26, 27]. We extracted HRs from the Kaplan-Meier survival curves in two of these studies [16, 26]. Significant heterogeneity was observed (*I*^*2*^ = 69.5%, *P* = 0.006), and a random effects model was used for the meta-analysis (Fig. 4). The pooled HR was 1.109, revealing that the patients with positive PD-L1 expression had a shorter PFS compared to those with negative PD-L1 expression; however, this difference was not statistically significant (95% CI: 0.581–2.117; *P* = 0.754).

### Association between PD-L1 expression and clinicopathological parameters

**Fig. 5:**
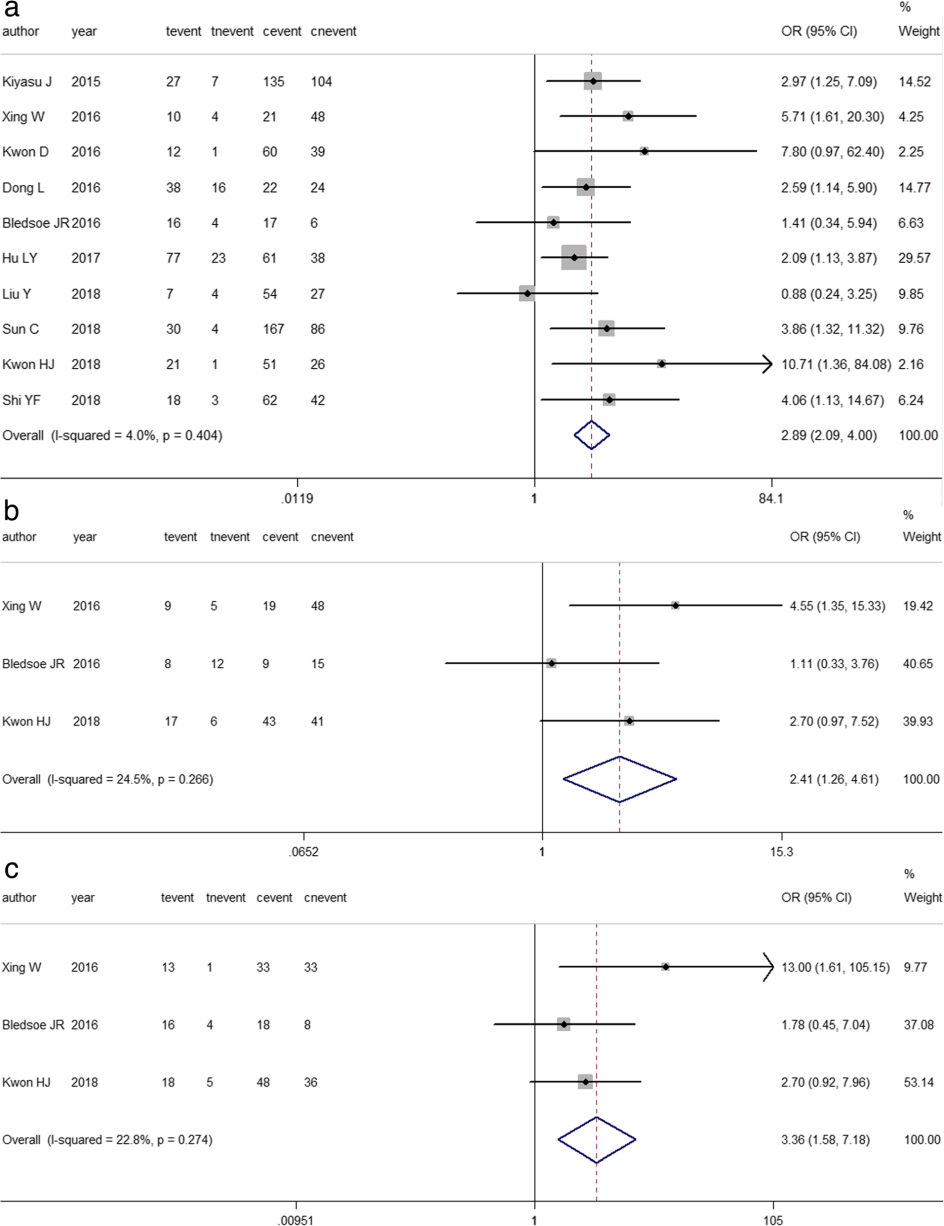
Forest plot describing the association between PD-L1 expression and pathological features. **a**. Pathological subtypes (t: non-GCB; c: GCB); **b**.BCL-6 (t: positive; **c**: negative); c.MUM-1 (t: positive; c: negative). event: positive PD-L1 expression; nevent: negative PD-L1 expression

**Fig. 6:**
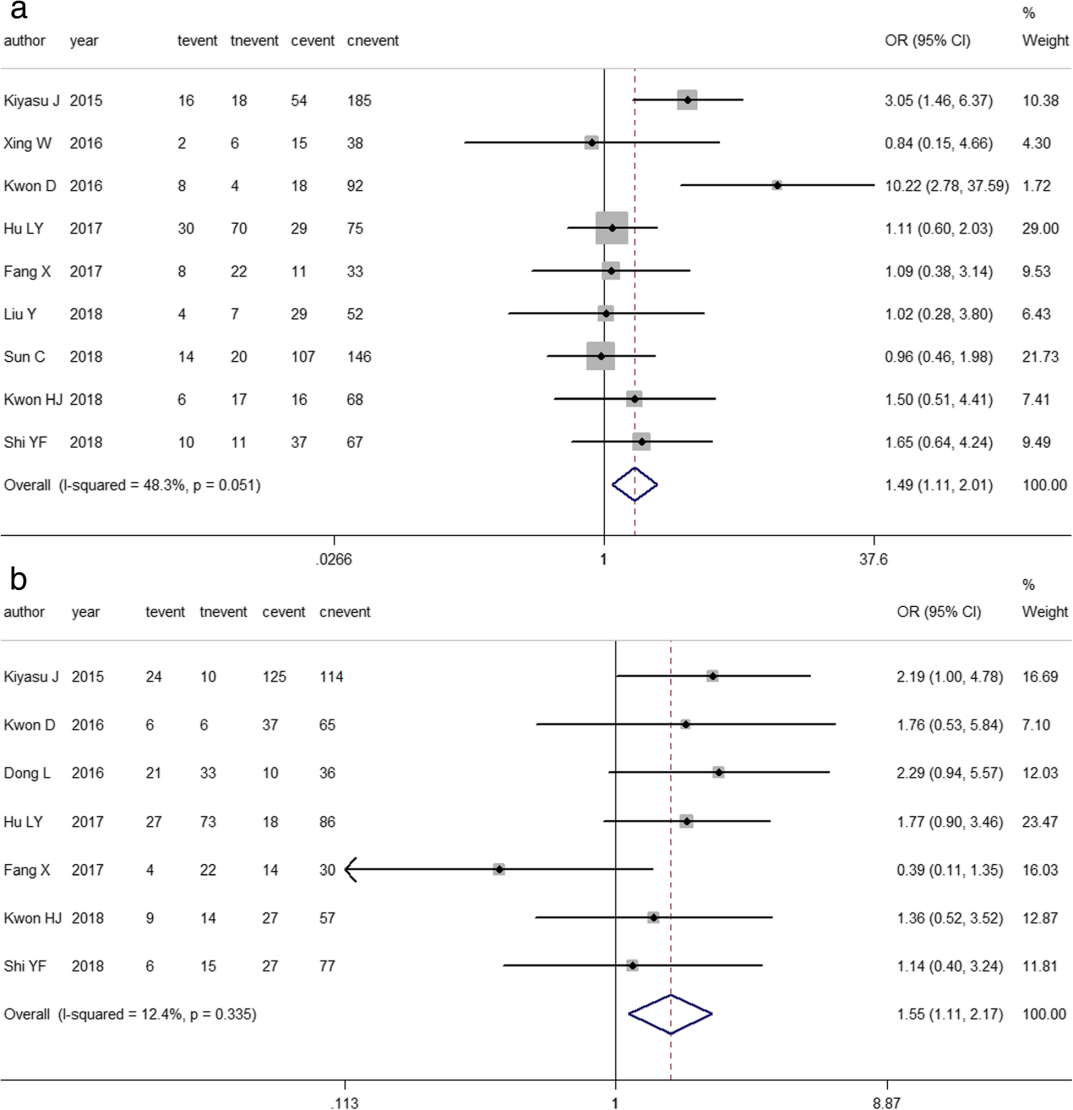
Forest plot describing the association between PD-L1 expression and clinical parameter features. **a**. B symptoms (t: yes; c: no). **b**. IPI score (t: 3–5; c: 1–2). event: positive PD-L1 expression; nevent: negative PD-L1 expression

Related studies were enrolled to analyze the association between PD-L1 expression and clinicopathological parameters. The results are listed in Table 3. DLBCL is divided into germinal center B-cell-like (GCB) and non-GCB subtypes according to the cell of origin [28]. Pooled ORs suggested that increased PD-L1 expression was significantly associated with the non-GCB subtype (OR = 2.891, 95% CI: 2.087–4.003, *P*<0.00; *I*^*2*^ = 4.0%, *P* = 0.404) [11, 16–18, 20, 21, 23, 25–27], negative expression of Bcl-6 (OR = 0.414, 95% Cl: 0.217–0.792, *P* = 0.008; *I*^*2*^ = 24.5%, *P* = 0.266) and positive expression of MUM1(OR = 3.365, 95% Cl: 1.578–7.175, *P* = 0.002; *I*^*2*^ = 22.8%, *P* = 0.274) [16, 20, 26] (Fig. 5a-c). For clinical parameters, 7 out of 13 articles showed that positive PD-L1 expression was more common with international prognostic index (IPI) scores of 3–5 in DLBCL patients (OR = 1.552, 95% CI: 1.111–2.169, *P* = 0.010; *I*^*2*^ = 12.40%, *P* = 0.335) [11, 17, 18, 21, 22, 26, 27]. Using a fixed effects model, a pooled OR from nine studies demonstrated that PD-L1 overexpression was related to B symptoms (OR = 1.495, 95% Cl: 1.109–2.015, *P* = 0.008; *I*^*2*^ = 48.3%, *P* = 0.051 [11, 16, 17, 21–23, 25–27]. (Fig. 6a-b).

**Table 3:** Association between PD-L1 expression and clinicopathological features

Clinicopathological parameters		No. of studies	No. of patients	Model	Pooled OR	95%CI	*P*	Heterogeneity	
								*I* ^*2*^	*P*
Age	≤60 vs. >60	12	1582	Fixed	0.887	0.675–1.166	0.390	0	0.744
Sex	female vs. male	11	1457	Fixed	1.068	0.819–1.392	0.626	9.50%	0.353
Pathology	GCB vs. non-GCB	10	1413	Fixed	2.891	2.087–4.003	0	4.00%	0.404
Stage	I-II vs. III-IV	9	1336	Fixed	1.209	0.915–1.599	0.182	26.40%	0.209
IPI	3–5 vs. 0–2	7	993	Fixed	1.552	1.111–2.169	0.010	12.40%	0.335
ECOG	≤1 vs. >1	7	1048	Random	1.006	0.445–2.274	0.988	64.40%	0.010
B symptom	no vs. yes	9	1345	Fixed	1.495	1.109–2.015	0.008	48.30%	0.051
CR	no vs. yes	5	632	Random	1.109	0.552–2.230	0.771	54.80%	0.065
LDH	normal vs. elevated	7	827	Fixed	1.341	0.939–1.915	0.107	25.50%	0.234
EB	no vs. yes	5	860	Random	2.180	0.485–9.799	0.309	72.20%	0.006
BCL-2	negative vs. positive	4	357	Fixed	1.510	0.857–2.661	0.154	0	0.776
BCL-6	negative vs. positive	3	232	Fixed	2.414	1.263–4.612	0.008	24.50%	0.266
CD10	negative vs. positive	3	233	Fixed	4.367	1.626–11.729	0.003	36.70%	0.206
MUM1	negative vs. positive	3	233	Fixed	3.365	1.578–7.175	0.002	22.80%	0.274
MYC	negative vs. positive	3	244	Fixed	1.252	0.647–2.420	0.504	0	0.627
ki-67%	low vs. high	4	397	Fixed	0.876	0.535–1.433	0.598	0	0.987

*IPI* international prognostic index; *ECOG* Eastern Cooperative Oncology Group; *GCB* germinal center B-cell-like; *OR* odd ratio; *LDH* Lactic dehydrogenase; *CR* complete remission

However, the associations were not significant between PD-L1 expression and age (≤60 vs.>60: OR = 0.887, 95% CI: 0.675–1.166, *P* = 0.39), gender (female vs. male: OR = 1.068, 95% CI: 0.819–1.392, *P* = 0.626), Ann Arbor stage(I-II vs. III-IV: OR = 1.209, 95% CI: 0.915–1.599, *P* = 0.182), Eastern Cooperative Oncology Group (ECOG) score (≤1 vs. > 1: OR = 1.006, 95% CI: 0.445–2.274, *P* = 0.988), lactic dehydrogenase (LDH) (normal vs. elevated: OR = 1.341, 95% CI: 0.939–1.915, *P* = 0.107), complete remission(CR)(no vs. yes: OR = 1.109, 95% CI: 0.552–2.230, *P* = 0.771), EB virus (EBV) in situ hybridization (negative vs. positive: OR = 2.180, 95% CI: 0.485–9.799, *P* = 0.309), Ki-67% (low vs. high: OR = 0.876, 95% CI: 0.535–1.433, *P* = 0.598), BCL2 (negative vs. positive: OR = 1.510, 95% CI: 0.857–2.661, *P* = 0.154) or MYC (negative vs. positive: OR = 1.252, 95% CI: 0.647–2.420, *P* = 0.504) (Table 3).

### Publication bias

**Fig. 7:**
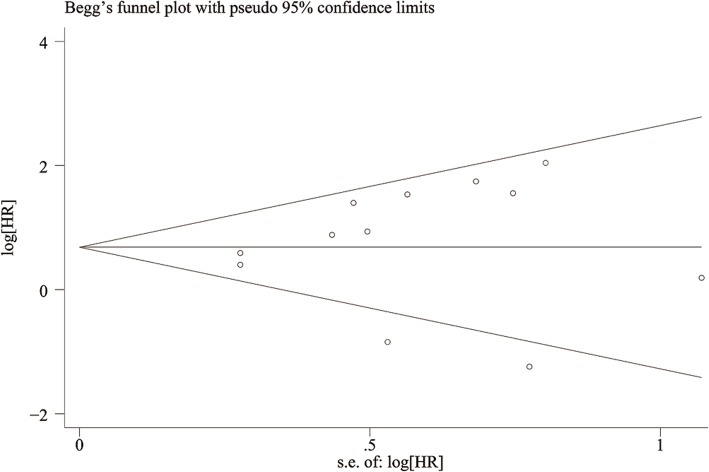
Funnel plot was constructed to visualize potential publication bias for D-L1 expression and OS

Begg’s test (*P* = 0.537) and Egger’s test (*P* = 0.586) demonstrated that there was no publication bias for positive PD-L1 expression regarding the HR of OS. Funnel plots revealed no publication bias for OS (Fig. 7).

## Discussion

Recently, investigators have focused more attention on PD-L1 expression in relation to the prognosis of various cancers. Structural variations disrupting the 3′ region of the PD-L1 gene and carcinogenic signal induction are potential mechanisms of elevated PD-L1 expression in many cancers, including DLBCL [4]. The number of studies researching the prognostic value of PD-L1 expression in DLBCL is limited, and the conclusions have been controversial. Many previous studies found that PD-L1 expression was related to worsened prognosis [11, 16, 18, 21, 22, 24, 25]. However, while some investigators found no prognostic significance of PD-L1 [19, 23, 26, 27], others demonstrated that PD-L1 was a favorable prognostic in DLBCL [17, 20]. This meta-analysis provided evidence to estimate the value of PD-L1 detection in tumor cells of DLBCL by pooling all related studies.

Our meta-analysis indicated that positive PD-L1 expression is significantly associated with inferior OS in DLBCL patients. The subgroup analyses demonstrated that PD-L1 expression was an indicator of poor prognosis in Asian populations, especially in Chinese individuals. One reason for these differences might be the genetic diversity among different ethnicities. In addition, we found that the prognostic role of PD-L1 in DLBCL demonstrated increased significance when the cutoff value was equal to or greater than 30%. This cutoff value could be beneficial for precisely stratifying a group of DLBCL patients with poorer outcomes. However, further research based on a large sample size is needed to confirm these speculations.

Most patients enrolled in our analysis received CHOP (cyclophosphamide, doxorubicin, vincristine and prednisone) or CHOP-like therapy, and more than half of them received rituximab at the same time. Three [11, 18, 25] of the included studies indicated that PD-L1 expression induced poor OS regardless of the use of rituximab. Hence, DLBCL patients with PD-L1 overexpression are unable to gain an increased benefit from the first-line therapy. Recently, an in vitro study of DLBCL cells (CRL2631) showed that the therapeutic effect of CHOP on decreasing the cell survival rate and increasing apoptosis was impaired when the PD-1/PD-L1 pathway was activated. However, this impairment would not occur without PD-L1 expression [29]. Therefore, one possible reason for the poor outcome in DLBCL with positive PD-L1 expression is that PD-L1 expression may contribute to chemotherapeutic resistance. A novel applicable strategy based on underlying molecular oncogenic mechanisms is necessary to treat these patients.

Our meta-analysis supports that PD-L1 could be a biomarker for poor outcome in DLBCL patients and is a highly promising therapeutic target. A phase Ib study including 11 relapsed/refractory DLBCL patients showed that an anti-PD-1 monoclonal antibody (nivolumab) could improve the objective response with a median follow-up time of 22.7 weeks [30]. In a phase II study, 66 DLBCL patients who received pidilizumab (anti-PD-L1) after autologous hematopoietic stem-cell transplantation (16-month PFS: 0.72) compared favorably with those not receiving pidilizumab after transplantation (18-month PFS: 0.52) from the same population [31].

However, in our study, positive PD-L1 expression in tumor cells had no significant impact on PFS. Possible reasons were that the number of studies about PFS was limited and that the HRs of PFS were extracted from the Kaplan-Meier curve in two studies [16, 26], which may compromise the precision of the data.

The relationship between PD-L1 expression and clinicopathological features was also assessed. We demonstrated support for a significant association between positive PD-L1 expression and non-GCB, which was aligned with previous researchers’ viewpoints [32, 33]. In addition, we found that positive PD-L1 expression was significantly associated both with positive MUM1 expression and negative BCL6 expression. The latter two expressive states were common in non-GCB based on the Hans classification. A possible mechanism of this tendency was that 9p24.1 amplification, leading to PD-L1 overexpression and JAK/STAT3 signaling activation, commonly occurred in non-GCB [34, 35]. Thus, anti-PD-1/PD-L1 may be more appropriate for patients with this aggressive subtype.

Our study also revealed that increased PD-L1 expression was more frequently observed with unfavorable clinical manifestations, including IPI score 3–5 and B symptoms. These results supported that positive PD-L1 expression might be beneficial for the identification of DLBCL patients with a higher risk of disease progression. However, more clinical trials are needed to demonstrate the adverse effects of PD-L1 expression in DLBCL.

Several studies have suggested that PD-L1 expression was associated with EBV infection [36, 37]. Chen et al. [32] included 16 EBV + DLBCL cases and all of them displayed positive PD-L1 expression, using a cutoff value of 0.05. A possible mechanism is that EBV-encoded latent membrane protein 1, or inflammatory factors, promote AP1- signaling and JAK/STAT signaling to activate PD-L1 enhancer and promoter regions [32, 38]. Despite this evidence, we found that the association between PD-L1 expression and EBV was not statistically significant, which might be due to limited data. This relationship needs to be further clarified.

Recently, considerable attention has been devoted to studies on DLBCL displaying various pathological features. Higher Ki-67 expression was regarded as indicative of higher proliferative activity of the lymphoma and as a robust predictor of poor prognosis in DLBCL patients with or without rituximab treatment [39]. The overexpression of MYC caused uncontrolled cell proliferation, while the antiapoptotic factor BCL2 was associated with drug resistance. It has been documented that MYC/BCL2 coexpression in DLBCL occurred more frequently in the non-GCB subtype and was associated with an aggressive clinical course [40]. However, our limited data demonstrated no significant association between these adverse prognostic biomarkers and PD-L1 expression, inferring that these biomarkers might affect disease processes through different signaling pathways. Further studies are needed to determine the interaction between PD-L1 and other pathological characteristics.

Several limitations should be acknowledged. First, the number of patients in the enrolled studies was relatively small, and most studies were performed in Asian populations. Thus, larger well-designed studies, especially in Western countries, are required to confirm these results. Second, the cutoff values defined by IHC varied in the included studies, which might cause increased heterogeneity in the final results. Therefore, establishing standardized definitions of positive PD-L1 expression using unified antibodies and detecting PD-L1 with other markers will likely enhance the reliability of the conclusion.

## Conclusion

Our meta-analysis supported that tumor cell PD-L1 expression in DLBCL patients was significantly associated with B symptoms, high IPI score (3–5), non-GCB subtype, positive MUM1 expression, negative BCL-6 expression and poor OS, which might be valuable for individual prognostic evaluations. Moreover, the results indicated that PD-1/PD-L1 might be a potential therapeutic target in DLBCL patients, especially for relapse/refractory cases. Further large-scale studies are warranted to clarify these findings and to assess the efficacy and safety of anti-PD-1 or PD-L1 therapy in DLBCL patients.

## Abbreviations

CHOP: cyclophosphamide, doxorubicin, vincristine and prednisone
CI: confidence interval
CR: complete remission
DLBCL: diffuse large B-cell lymphoma
EBV: EB virus
ECOG: Eastern Cooperative Oncology Group
GCB: germinal center B-cell-like
HL: Hodgkin lymphoma
HR: hazard ratio
IHC: immunohistochemistry
IPI: international prognostic index
LDH: lactic dehydrogenase
NA: not available
NHL: non-Hodgkin lymphoma
OR: odds ratio
OS: overall survival
PD-1/PD-L1: programmed cell death receptor 1 /ligand 1
PFS: progression-free survival
R: rituximab
RT: radiotherapy
